# The impact of risk prioritization in COVID-19 vaccination in Belgium on hospital and intensive care unit admissions

**DOI:** 10.1093/eurpub/ckaf202

**Published:** 2025-11-01

**Authors:** Elias Vermeiren, Toon Braeye, Veerle Stouten, Jan De Maeseneer, Charlotte Scheerens, John Crombez, Joris A F van Loenhout

**Affiliations:** Department of Epidemiology and Public Health, Sciensano, Brussels, Belgium; Department of Epidemiology and Public Health, Sciensano, Brussels, Belgium; Department of Epidemiology and Public Health, Sciensano, Brussels, Belgium; Department of Public Health and Primary Care, Ghent University, Ghent, Belgium; WHO Collaborating Center on Family Medicine and Primary Health Care, Ghent University, Ghent, Belgium; Department of Public Health and Primary Care, Ghent University, Ghent, Belgium; UNU-CRIS, Bruges, Belgium; Department of Public Health and Primary Care, Ghent University, Ghent, Belgium; Ghent University Hospital, Ghent, Belgium; Department of Epidemiology and Public Health, Sciensano, Brussels, Belgium

## Abstract

Belgium prioritized primary COVID-19 vaccination of persons aged 18–64 years with underlying health conditions over their same-aged peers, leading to an accelerated administration of a first dose of 35 days. We assessed this strategy’s impact on hospital admissions, in comparison to alternative scenarios, using generalized causal inference techniques with a hierarchical Bayesian model. A solely age-based scenario showed a significant increase in hospital admissions of 254, while random-allocation and no-vaccination scenarios showed even higher increases (604 and 1998, respectively). These results emphasize the importance of prioritization strategies in a pandemic context and the benefits of COVID-19 vaccines in general.

## Introduction

When COVID-19 vaccines became available in early 2021, strategies were developed to maximize their public health impact. Age, having comorbidities, and essential worker status were the most important risk factors used to determine vaccination prioritization. Age and having comorbidities are risk factors for severe COVID-19, and essential workers were the most likely to get infected [1]. Belgium’s national Task Force Vaccination initially prioritized residents and staff from care institutions (e.g., nursing homes, hospitals). The further roll-out continued based on age, from oldest to youngest, for persons aged 65 years and over. For those aged 18–64 years, age-based prioritization was complemented with risk-based prioritization: individuals with underlying health conditions associated with an increased risk of severe COVID-19 were prioritized from the second of April 2021 onwards [2]. A previous study reported that risk-prioritized persons received their first vaccine dose 35 days earlier than those of the same age without risk-prioritization [3].

In this study, we aimed to assess whether risk-prioritization reduced severe outcomes of COVID-19 by comparing hospital and intensive care unit (ICU) admissions during vaccine roll-out to those simulated under alternative vaccine-allocation scenarios.

## Methods

Our cohort consisted of the Belgian population aged 18–64 eligible for vaccination between 2 April and 8 July 2021, excluding healthcare workers who received earlier vaccination. We estimated the number of hospital admissions during this period with generalized causal inference using a hierarchical Bayesian model to simultaneously estimate admission probabilities and the effects of different allocation scenarios. Person-level data for the model fit and Belgium’s weekly number of COVID-19 admissions were obtained from data sources previously described [3].

The probability of admission by age (18–24, 25–34, 35–44, 45–54, and 55–64 years), risk-prioritization, vaccination status (defined as having received at least one dose or being unvaccinated), and calendar week was estimated using Binomial regression. All coefficients had non-informative, normally distributed priors.

We simulated vaccine allocation under three scenarios. In two scenarios, we maintained the observed weekly vaccination rates and final coverage. The ‘age-based scenario’ removed risk-prioritization while preserving age-based allocation proportional to the distribution observed in non-risk-priority groups. The ‘random-allocation scenario’ eliminated both risk- and age-based prioritization. A ‘no-vaccination scenario’ served as a third comparator to estimate overall vaccine impact. A detailed description of our methods—including model specification, variables, fitting procedures, and R code—is available in the supplementary materials.

## Results

Over the study period 2697 hospital and 459 ICU admissions were reported through the CHS and linked to person-data on vaccination and prioritization. The participation in the CHS declined: at the start of the study period, 65% of admissions were reported, compared to 38% at the end.

**Figure 1.**
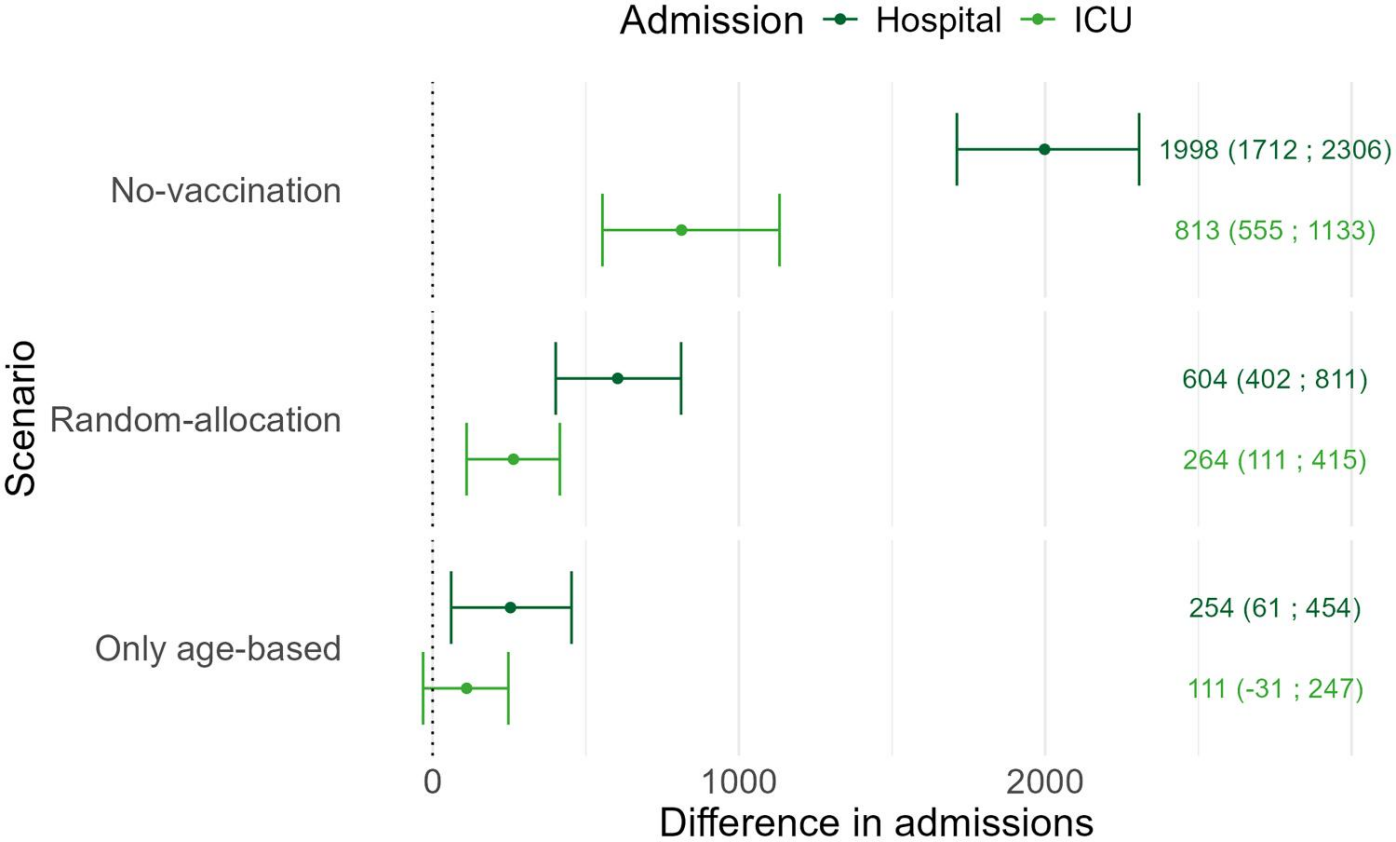
The difference in hospital and ICU admissions between the actual implemented age-based and risk-prioritized vaccination strategy in Belgium and three simulated scenarios. The points depict the difference between the actual and each simulated scenario. The vertical bars show 95% credibility intervals. The results are coloured by admission type. No-vaccination scenario: 1998 (95%CrI 1712 to 2306) hospital and 813 (95%CrI 555 to 1133) ICU admissions; random-allocation scenario: 604 (95%CrI 402 to 811) hospital and 264 (95%CrI 111 to 415) ICU admissions; only age-based scenario: 254 (95%CrI 61 to 454) hospital and 111 (95%CrI –30 to 247) ICU admissions.

A solely age-based vaccination scenario resulted in an additional 254 (95% Credibility Interval (CrI) 61 to 454) hospital and 111 (95%CrI –30 to 247) ICU admissions compared to risk-prioritization (Fig. 1). Random-allocation resulted in an additional 604 (95%CrI 402 to 811) hospital and 264 (95%CrI 111 to 415) ICU admissions, while no-vaccination resulted in an additional 1998 (95%CrI 1712 to 2306) hospital and 813 (95%CrI 555 to 1133) ICU admissions.

## Discussion

We estimated that additionally including risk-prioritization in an age-based vaccination strategy averted 254 hospital admissions during the 14 weeks of vaccine roll-out to 18–64 year-olds in Belgium. The reduction in ICU admissions was not statistically significant, possibly due to the relatively small number of observations. We found higher numbers of averted hospital and ICU admissions for scenarios with random allocation or without vaccination, further evidencing the importance of prioritization strategies and the benefits of COVID-19 vaccines in general.

Previous research on COVID-19 vaccine allocation has primarily focused on balancing between the reduced transmission associated with prioritizing younger individuals and the direct protection from severe outcomes associated with prioritizing older individuals [4]. While research has also considered essential workers, spatial heterogeneity and vaccine interval timing, the search for the optimal allocation strategy has predominantly centred around age-based prioritization. Despite recommendations to perform risk-prioritization (e.g. WHO [5]), few studies have considered the impact of additional risk-based prioritisation [1, 6, 7]. Our result, a small but significant reduction in the number of admissions, agrees with this prior research. Islam *et al.* [6]considered prioritizing risk to have additional benefits over a strategy based only on age. Moore *et al.* [7] state that an ‘ideal program would involve an ordering of each age group split into those with and without comorbidities, though in practice this would likely be too complex to implement’. They predicted the results of such an additional risk-prioritization to be relatively small given the overlap between risk and age. Chapman *et al.* [1] proclaimed that age-based prioritization yielded the best outcome, compared to only comorbidity, essential worker or special population (prisoners and the homeless) prioritization in averting deaths. They state that considering multiple risk factors in vaccine prioritization may attribute to an additional benefit in averted outcome; however, feasibility and policy must be balanced.

While compartmental models have been dominant in this research, we opted for a causal inference approach. This more straightforward approach cannot account for scenario-specific differences in transmission, but does also not require data or assumptions on the differences in behaviour by risk group before and after vaccination or on the link between vaccine hesitancy and prioritization [8]. The complexity of compartmental models is both a strength and a weakness. Indirect effects can be included in the evaluation, but results are often less transparent and conditional on many parameters. This is made clear by the continued discussion on age-based prioritization [4, 9]. Likewise, our approach has strengths and weaknesses. Our simulated scenarios were set up to mirror reality; the no-vaccination scenario aside, we only varied the order or priority of vaccine administration. Other variables remained constant and reflected observed constraints in supply, deployment capacity, and vaccine acceptance. Our work should be interpreted given the public health and social measures and the speed of vaccine roll-out during the spring of 2021. This resulted in a study period that was relatively short, during which the overall virus circulation was low. Additional limitations to our study are that other outcomes, such as mortality, remain largely unexplored. The included data are not exhaustive. We estimated to have included 85% of the Belgian population and between 38% and 65% of hospital admissions. These limitations have been investigated elsewhere [10]. They will limit the precision of our estimates and might have introduced bias. While we have no indication of selective reporting associated with either vaccination or risk profiles, we cannot exclude it either. In conclusion, our findings support the use of a combination of risk- and age-based prioritization. Protecting those at higher risk earlier will maximize the impact of vaccination during vaccine roll-out.

## Supplementary Material

ckaf202_Supplementary_Data

## Data Availability

As the LINK-VACC project is not an open-access platform. the individual level data are only available to researchers working on the project. However, general descriptive statistics from the registries used in LINK-VACC are available from https://epidata.sciensano.be/epistat/dashboard/#covid. This includes inter alia the number of confirmed cases by date, age, sex and province or the number of administered vaccines by date, region, age, sex, brand and dose.
