# Supplementary material for: The impact of risk prioritization in COVID-19 vaccination in Belgium on hospital and intensive care unit admissions
Source: Eur J Public Health. 2025 Nov 1;36(3):ckaf202. doi: 10.1093/eurpub/ckaf202 (PMC13230501; doi:10.1093/eurpub/ckaf202)
Supplement: ckaf202_Supplementary_Data [file ckaf202_supplementary_data.docx]

Method S1

###### In-detail methodology for “The impact of risk prioritisation in COVID-19 vaccination in Belgium on hospital and ICU admissions”

In this section we presented a more detailed description of the methodology used and we included the R-code for the nimble model. Within the model, three things were simultaneously estimated: (1) the proportion of intakes in the CHS compared to the SCS (2) the relation between vaccination, prioritization, age and hospital admission probability and (3) predictions for the number of hospital admissions given alternative vaccine allocation scenarios.

## Admission data

Two surveys collecting hospital admission data were used: The clinical hospital survey (CHS) and the Surge Capacity Survey (SCS). The CHS contains person-level records. Hospital participation was voluntary. Within the CHS we could differentiate admissions of prioritized and non-prioritized persons. The SCS contains hospital-level records. Hospital participation was mandatory. From the SCS, we could obtain the total number of admissions over a given period.

Since, given the data, we can only estimate the probability of admission to the CHS, we need a proportion (intakes in CHS per week over intakes in SCS per week) to translate this probability to an overall probability of hospital admission.

Previous analyses already indicated representativeness of the CHS sample [1]. The proportion was given a neutral noninformative prior: Beta(1/3,1/3).

Person-level data was further obtained by linking pseudonymised hospital admission records from the CHS to an exhaustive population and vaccination registry and a database indicating the risk-stratification (binary, yes/no risk-priority). Additional details on the data can be found elsewhere [2,3].

## A model for the probability of hospital admission

We estimated the adjusted probability of hospital admission using a hierarchical Bayesian model. The model's core is a binomial regression with a probit link function. The selection of predictors was intentionally parsimonious, including age (age groups-, risk-prioritization status (binary), vaccination status (binary), and calendar week (14 factor-levels) as factorial main effects. An interaction between age and risk-prioritization was included based on model fit, as assessed by the Bayesian Information Criterion (BIC). This parsimonious approach was chosen to estimate the total associative effect of the risk-prioritization policy. Our goal was to predict outcomes for the real-world group of prioritized individuals, who are defined by a mix of biological and social risk factors. We, therefore, intentionally did not adjust for potential upstream common causes (e.g., socioeconomic status), which would be necessary for estimating a direct etiological effect but is inappropriate for evaluating the full impact of a policy targeting this specific group.

The priors for the intercept and all factor-level coefficients were specified as non-informative, wide normal distributions: Normal(0, precision=0.0001).

$$Probit\left( P_{admission} \right) \sim age + prioritization + age*prioritization+vaccination + week$$

As study results, we presented the posterior distributions of intake differences between scenarios. The CHS also detailed the evolution of the patient after hospital admission (including ICU admission). The same modelling approach was applied separately for the ICU admission outcome.

## The Scenarios

Once the model estimates the admission probability for every population group, we can predict the number of hospitalizations for different vaccination allocation scenarios. We do this by multiplying the population count in each group by its estimated admission probability. Each scenario is defined by a matrix (referred to in the code as numWeek.age), which specifies the number of vaccinated and unvaccinated people in each group (a combination of age, risk-priority and vaccination) for each week.

Two alternative allocation scenarios were ‘age based’ and ‘random’ allocation. Their matrices were built on two key constraints based on the real-world rollout:

**Constant Vaccine Supply:** The total number of new vaccines administered each week is identical across all scenarios.

**Constant Final Coverage:** All scenarios end with the same total number of people vaccinated in each group by the final week.

The scenarios only differ in who receives the vaccine each week. For example, over time, the number of people in an unvaccinated group decreases while the number of people in vaccinated groups increases, but the sum remains constant.

For the ‘no vaccination’ scenario, the numbers in the vaccinated groups remained at zero.

## Scenario example: age-based

In the 'age-based’ scenario, we simulated a rollout where, within an age group, both risk and non-risk individuals are vaccinated at the same proportional rate. The relative vaccination speed between different age groups is based on what was observed in the non-prioritized population. We illustrate this with a simplified (we reduced the number of week and the number of age groups) and fictional numbers-example. As the actual ‘age based’ scenario two numbers are kept constant: the final coverage achieved and the number of vaccines administered each week.

First we present the observed numbers in our illustrative example. By week 3, the end of this example’s study period, there are 600 vaccinated persons in age group 1 (AG1) 400 without additional risk priority and 200 with additional risk priority.

| Category | AG1/No Risk/Vaccinated (N=600) | AG1/Risk/Vaccinated (N=250) | AG2/No Risk/Vaccinated (N=700) | AG2/Risk/Vaccinated (N=300) |  |
| --- | --- | --- | --- | --- | --- |
| Week 1 | 20 | 100 | 0 | 40 |  |
| Week 2 | 200 | 180 | 100 | 160 |  |
| Week 3 | 400 | 200 | 400 | 200 |  |

To have an alternative solely age-based scenario for this example, we keep the final row. In addition, we still administer 160 vaccines in the first week and 480 in the second week. To differentiate between age groups we only consider the observed ‘no risk-priority’ groups. In these ‘no-risk priority groups’ all vaccines administered in week 1 were administered to AG1. Therefore all 160 vaccines would be administered to AG1. Given that we want to obtain a final number of 400 (No risk) and 200 (Risk) vaccines administered at the same speed 107 (two thirds of the 160 available vaccines) vaccines will be administered to the ‘no-risk priority’ group and 53, one third of the available vaccines, to the ‘risk priority’ group. In the second week we need to administer 480 vaccines, 9 out of 14 vaccines were administered to AG1, 5 out of 14 to AG2, (as observed in the ‘no-risk priority’ group).

| Category | AG1/No Risk/Vaccinated (N=600) | AG1/Risk/Vaccinated (N=250) | AG2/No Risk/Vaccinated (N=700) | AG2/Risk/Vaccinated (N=300) |  |
| --- | --- | --- | --- | --- | --- |
| Week 1 | 107 | 53 | 0 | 0 |  |
| Week 2 | 313 | 156 | 114 | 57 |  |
| Week 3 | 400 | 200 | 400 | 200 |  |

From this fictional, simplified example, it is clear that in an age-based scenario AG1 continues to be vaccinated faster than AG2 (as observed), but the risk-priority subgroups experience slower vaccination as they are no longer prioritized for vaccination.

## MCMC fit

The model was fitted using MCMC using the default nimble sampler. We ran three chains for 100,000 iterations each, discarding the first 30,000 as burn-in and applying a thinning interval of 100. Convergence was confirmed using the Gelman-Rubin diagnostic and through visual inspection of trace plots.

## R code

VP.code <-nimbleCode({

# colIdx was added as column enumerable, this represents each age group

# all variables that looped through the weeks got an extra dimension (matrix)

# all vars that were constant, were vectorized to represent each age group

##########################################################################

# Likelihoods: (1) proportion CHS/SCS by week (2) Observed hosp by group

##########################################################################

for(rowIdx in 1:n.row){

chsWeek[rowIdx] ~ dbin(rel.to.abs.wk[rowIdx], scsWeek[rowIdx])

rel.to.abs.wk[rowIdx] ~ dbeta(1/3, 1/3)

}

for(colIdx in 1:n.col){

for(rowIdx in 1:n.row){

chsWeek1[rowIdx,colIdx] ~ dbin(prob.chs[rowIdx,colIdx], numWeek[rowIdx,colIdx])

# Lin Pred to Probit to estimte prob.chs

probit(prob.chs[rowIdx,colIdx]) <- coefInt + coefAge[age[colIdx]] + coefVacc[vacc[colIdx]] +

coefPrio[prio[colIdx]] + coefWeek[calWeek[rowIdx]] +

coefAgePrio[ageprio[colIdx]]

# Translate CHS to SCS

prob.scs.wk[rowIdx,colIdx] <- prob.chs[rowIdx,colIdx]/rel.to.abs.wk[rowIdx]

# Sample from posterior (to assess model fit)

intakes.chs[rowIdx, colIdx] ~ dbin(prob.chs[rowIdx,colIdx], numWeek[rowIdx,colIdx])

intakes.scs[rowIdx, colIdx] ~ dbin(prob.scs.wk[rowIdx,colIdx], numWeek[rowIdx,colIdx])

average.intakes.scs[rowIdx, colIdx] <- prob.scs.wk[rowIdx,colIdx]*numWeek[rowIdx,colIdx]

extrapol.intakes.scs[rowIdx, colIdx] <- intakes.chs[rowIdx, colIdx]/rel.to.abs.wk[rowIdx]

############ Scenarios

# No prioritization, only age

average.intakes.scs.age[rowIdx, colIdx] <- prob.scs.wk[rowIdx,colIdx]*numWeek.age[rowIdx,colIdx]

# Novax

average.intakes.scs.novax[rowIdx, colIdx] <- prob.scs.wk[rowIdx,colIdx]*numWeek.novax[rowIdx,colIdx]

# Random

average.intakes.scs.R[rowIdx, colIdx] <- prob.scs.wk[rowIdx,colIdx]*numWeek.R[rowIdx,colIdx]

}

# Sum over rows to have sample of intakes per group

intakes.chs.col[colIdx] <- sum(intakes.chs[1:n.row, colIdx]) # simulated original intake

intakes.scs.col[colIdx] <- sum(intakes.scs[1:n.row, colIdx]) # simulated original intake

average.intakes.scs.col[colIdx] <- sum(average.intakes.scs[1:n.row, colIdx])

extrapol.intakes.scs.col[colIdx] <- sum(extrapol.intakes.scs[1:n.row, colIdx])

# No prioritization, only age

average.intakes.scs.age.col[colIdx] <- sum(average.intakes.scs.age[1:n.row, colIdx])

# Novax

average.intakes.scs.novax.col[colIdx] <- sum(average.intakes.scs.novax[1:n.row, colIdx])

# Random

average.intakes.scs.R.col[colIdx] <- sum(average.intakes.scs.R[1:n.row, colIdx])

}

intakes.chs.all <- sum(intakes.chs.col[1:n.col]) # simulated original intake

intakes.scs.all <- sum(intakes.scs.col[1:n.col]) # simulated original intake

average.intakes.scs.all <- sum(average.intakes.scs.col[1:n.col]) # simulated original intake

p.average.intakes.scs.all <- average.intakes.scs.all/total_pop

extrapol.intakes.scs.all <- sum(extrapol.intakes.scs.col[1:n.col])

p.extrapol.intakes.scs.all <- extrapol.intakes.scs.all/total_pop

# intakes.chs.age.all <- sum(intakes.chs.age.col[1:n.col])

# intakes.scs.age.all <- sum(intakes.scs.age.col[1:n.col])

average.intakes.scs.age.all <- sum(average.intakes.scs.age.col[1:n.col])

p.average.intakes.scs.age.all <- average.intakes.scs.age.all/total_pop

# Novax

average.intakes.scs.novax.all <- sum(average.intakes.scs.novax.col[1:n.col])

p.average.intakes.scs.novax.all <- average.intakes.scs.novax.all/total_pop

# Random

average.intakes.scs.R.all <- sum(average.intakes.scs.R.col[1:n.col])

p.average.intakes.scs.R.all <- average.intakes.scs.R.all/total_pop

# Difference Number of intakes (extrapolated) and expected in the different scenarios

diff.obs.scs.age <- extrapol.intakes.scs.all-average.intakes.scs.age.all

diff.obs.scs.R <- extrapol.intakes.scs.all-average.intakes.scs.R.all

diff.obs.scs.novax <- extrapol.intakes.scs.all-average.intakes.scs.novax.all

# Percentage increase of different scenarios

perc.inc.obs.scs.age <- average.intakes.scs.age.all/extrapol.intakes.scs.all

perc.inc.obs.scs.R <- average.intakes.scs.R.all/extrapol.intakes.scs.all

perc.inc.obs.scs.novax <- average.intakes.scs.novax.all/extrapol.intakes.scs.all

#########

# Priors

#########

coefAge[1] <- 0

for(iAge in 2:n.age){

coefAge[iAge] ~ dnorm(0,0.0001)

}

coefPrio[1] <- 0

for (iPrio in 2:n.prio){

coefPrio[iPrio]~dnorm(0,0.0001)

}

coefVacc[1] <- 0

for (iVacc in 2:n.vacc){

coefVacc[iVacc]~dnorm(0,0.0001)

}

coefWeek[1] <- 0

for(iWeek in 2:n.row){

coefWeek[iWeek] ~ dnorm(0.0000,0.0001)

}

coefAgePrio[1] <- 0

for(iAgePrio in 2:n.ageprio){

coefAgePrio[iAgePrio] ~ dnorm(0.0000,0.0001)

}

coefInt ~ dnorm(0,0.0001)

})
